# The application of the palliative prognostic index, charlson comorbidity index, and Glasgow prognostic score in predicting the life expectancy of patients with hematologic malignancies under palliative care

**DOI:** 10.1186/s12904-015-0011-5

**Published:** 2015-04-30

**Authors:** Wen-Chi Chou, Chen-Yi Kao, Po-Nan Wang, Hung Chang, Hung-Ming Wang, Pei-Hung Chang, Kun-Yun Yeh, Yu-Shin Hung

**Affiliations:** Division of Hematology-Oncology, Department of Internal Medicine, Chang Gung Memorial Hospital Linkou branch, and School of Medicine, Chang Gung University, No. 5 Fuxing Street, Guishan Township, Taoyuan Taiwan; Division of Hematology-Oncology, Department of Internal Medicine, Chang Gung Memorial Hospital Keelung branch, Keelung, Taiwan

**Keywords:** Palliative prognostic index, Charlson comorbidity index, Glasgow prognostic score, Prognostication, Hematologic malignancy, Palliative care

## Abstract

**Background:**

The clinical course for hematologic malignancy varies widely and no prognostic tool is available for patients with a hematologic malignancy under palliative care. To assess the application of the Palliative Prognostic Index (PPI), Charlson Comorbidity Index (CCI), and Glasgow Prognostic Score (GPS) as prognostic tools in patients with hematologic malignancies under palliative care.

**Methods:**

We included 217 patients with pathologically proven hematologic malignancies under palliative care consultation service (PCCS) between January 2006 and December 2012 at a single medical center in Taiwan. Patients were categorized into subgroups by PPI, CCI, and GPS for survival analysis.

**Results:**

The median survival was 16 days (interquartile range, 4–47.5 days) for all patients and 204 patients (94%) died within 180 days after PCCS. There was a significant difference in survival among patients categorized using the PPI (median survival 49, 15, and 7 days in patients categorized into a good, intermittent, and poor prognostic group, respectively) and the GPS (median survival 66 and 13 days for GPS 0 and 1, respectively). There was no difference in survival between patients with a GPS score of 0 versus 2, or a CCI score of 0 versus ≥1. The survival time was significantly discriminated after stratifying patients with a good PPI score based on the CCI (median survival 102 and 41 days in patients with a CCI score of 0 and ≥1, respectively) from those with a poor PPI score by using the GPS (median survival 47 and 7 days in patients with GPS scores of 0 and 1–2, respectively).

**Conclusions:**

PPI is a useful prognosticator of life expectancy in terminally ill patients under palliative care for a hematologic malignancy. Concurrent use of the GPS and CCI improved the accuracy of prognostication using the PPI.

## Background

Hematologic malignancies are distinct from solid cancers; they are characterized by disseminated tumor involvement and usually present with symptoms related to bone marrow failure such as infection, bleeding, and anemia, rather than direct compression by a solid tumor [1]. Hematologic malignancies are also relatively rare, accounting for less than 5% of total cancer deaths in Taiwan annually [2].

Several prognostic scores for predicting patient survival have been developed, based on clinical features, associated comorbidity, and laboratory data. Three of most widely used scores for predicting life expectancy of cancer patients are the Palliative Prognostic Index (PPI) [3-6], the Charlson Comorbidity Index (CCI) [7-12], and the Glasgow Prognostic Score (GPS) [13]. The PPI is used to predict life expectancy in terminally ill cancer patients under palliative care and is scored by presentation of clinical features on the day of palliative care referral [3]. The PPI has been validated in different hospice settings without reference to a particular cancer type [4-6]. The application of the PPI as a prognosticator in patients with a given cancer type is uncertain. The CCI is based on 1-year mortality data from internal medicine patients admitted to an inpatient setting and is the most widely used comorbidity index in cancer patients [7,8]. The CCI has been widely used as a predictor and validated for patients with hematologic malignancies and who are undergoing antitumor therapy [9-12]. The role of the CCI in the palliative care setting has not been investigated. GPS, combining serum C-reactive protein (CRP) and albumin levels, is an inflammation based-prognostic score and has been demonstrated to have independent prognostic for solid cancer patients with operative or inoperative disease, and those undergoing chemotherapy [13]. However, there is little data regarding the role of GPS as a prognosticator in patients with hematologic malignancy.

Because no prognostic tool was available for patients with a hematologic malignancy under palliative care, we retrospectively analyzed the clinical characteristics of these patients, who were under the palliative care consultation service (PCCS) at a medical center in Taiwan. We wished to assess the application of the PPI, CCI, and GPS as prognostic tools in terminally ill patients with a hematologic malignancy under the care of PCCS.

## Methods

### Patient selection

A cohort of 4685 terminally ill cancer patients who received care from PCCS between January 2006 and December 2011 at a single medical center in Taiwan (Chang Gung Memorial Hospital at Linkou) was recorded in our database. All the patients were referred to PCCS because their clinician judged that they would benefit from palliative care and were unlikely to survive longer than 6 months. Among these patients, 230 (4.9%) had a pathological-proven hematologic malignancy and were included in this study. Thirteen patients were excluded because of an incomplete record of GPS components, and the remaining 217 patients were enrolled for survival analysis. The study protocol was ed by the Institutional Review Board of the Chang Gung Memorial Hospital (#101-1980B), in compliance with the Helsinki Declaration (1996). Written informed consent was obtained from all patients before receiving the PCCS.

### Data collection

Patient demographics, including age, gender, and diagnoses of hematologic malignancy (lymphoma, leukemia or multiple myeloma) were recorded at the first consultation by a specialist nurse using a formulated “patient record form” developed by the Bureau of Health Promotion and based on the clinical experience of terminal cancer in Taiwan [14]. The “patient record form” consisted of the Eastern Cooperative Oncology Group performance status (ECOG PS) and 29 distress symptoms assessed by the patient including dyspnea, edema, delirium, and loss of appetite, as well as the amount of food and fluids they had taken orally. The physical symptoms of patients were recorded by a palliative care physician or nurse specialist using the same form at each PCCS visit. Patients were evaluated to establish the presence or absence of each physical symptom. If patients were receiving total parental nutrition or had an enteral feeding tube, they were included in a “normal” oral intake category. Delirium was diagnosed using the criteria of the Diagnostic and Statistical Manual of Mental Disorders (Fourth Edition). For patients who had difficulty with verbal communication, a nurse specialist assessed their status using a proxy or caregiver response.

The PPI is the sum of the Palliative Performance Scale (PPS) [15] and scores for 4 other clinical variables: oral intake, edema, resting dyspnea, and delirium, giving a number between 0 and 15. The PPI was calculated for each patient using the “patient record form” filled in on the day that PCCS took over their care. To simplify PPI calculations, we used the ECOG PS instead of the PPS, where the ECOG PS scale scores of 0–2, 3, and 4 corresponded to PPS scores of 100–60, 30–50, and 10–20, respectively [16]. Any comorbidity when PCCS took over care were obtained retrospectively from the patient’s electronic chart. A modified CCI, which has been validated for patients with hematologic malignancies, was scored according to the patient’s comorbidity [9]. The CRP and albumin levels within 14 days of the patient’s care being taken over by PCCS were retrospectively obtained from the electronic chart and were used to calculate the GPS. The survival time was defined from the first day of patient care by PCCS to the day of death. For outpatients, the date of death was obtained from either the cancer registration center in our institute or the National Register of Death Database in Taiwan. Surviving patients were censored 180 days from the first day of PCCS referral.

### Statistical analysis

Basic demographic data were summarized as n (%) for categorical variables, and the median with the interquartile range (IQRs, 25–75%) for continuous variables, respectively. Patients were stratified into subgroups on the basis of the PPI, GPS, and CCI for survival analysis. For the PPI, patients were divided into good (score 0–4), intermediate (score 4.5–6) and poor prognostic groups (score >6) according to the PPI score, using the same categories as our previous reports [6]. Patients were divided into good (score 0), intermediate (score 1), and poor prognostic groups (score 2) on the basis of GPS. Due to the wide variation in the CCI among our patients (range, 0–12; IQR, 0–2), they were divided into good (CCI = 0) and poor prognostic groups (CCI ≥ 1), using the optimal cut-off categories of the CCI, which were selected with the intent of generating preliminary data with a better intra-group difference in survival time. Patients in different PPI groups were further stratified by their GPS and CCI scores with the intention to evaluate their prognostic value in each different PPI group. Overall survival was calculated using the Kaplan-Meier method. Log-rank tests were used to determine the significant differences between the survival curves. Hazard ratios (HRs) for subgroup categories with respect to the PPI, GPS, and CCI were estimated using multivariate Cox regression after adjusting for age, gender, and type of hematologic malignancy, which were also adjusted to minimize bias from care received from other sources. Statistical analyses were performed using SPSS 17.0 statistics software (SPSS Inc, Chicago, IL, USA). All statistical assessments were considered significant when *P* < 0.05.

## Results

Table 1 shows the patient demographic data, and the prevalence rate of core components that constitute the PPI, GPS, and CCI of the 217 patients. The median patient age was 63.3 years (IQR, 46–76 years) and 61.3% of the patients were men. Acute leukemia was the most common disease (131 patients, 60.4%), followed by lymphoma (66 patients, 30.4%) and multiple myeloma (20 patients, 9.2%). The median survival time was 16 days (IQR, 4–47.5 days). At the end of the follow up period, 204 patients (94%) had died, and 13 patients (6%) had survived for more than 180 days.

**Table 1:** **Baseline patient demographic data**

**Patient characteristics (n = 217)**	**Number (%)**
Age (years), median (IQR)	63.3 (46–76)
<40	48 (22.1)
40-75	110 (50.7)
>75	59 (27.2)
Gender	
Male	133 (61.3)
Female	84 (38.7)
Origin of primary tumor	
Leukemia	131 (60.4)
Lymphoma	66 (30.4)
Multiple myeloma	20 (9.2)
Core components and scoring of PPI	
ECOG performance status 0–2 (score 0)	36 (16.6)
ECOG performance status 3 (score 2.5)	58 (26.7)
ECOG performance status 4 (score 4)	123 (56.7)
No dyspnea (score 0)	91 (41.9)
Dyspnea at rest (score 3.5)	126 (58.1)
No delirium (score 0)	187 (86.2)
Delirium present (score 4)	30 (13.8)
No edema (score 0)	168 (77.4)
Edema present (score 1)	49 (22.6)
Oral intake normal (score 0)	115 (53.0)
Oral intake reduced but more than one mouthful (score 1)	87 (40.1)
Oral intake less than one mouthful (score 2.5)	15 (6.9)
Total PPI score, median (IQR)	7 (4–8.5)
Components and scoring of GPS	
CRP >10 (score 1)	181 (83.4)
Albumin <3.5 (score 1)	156 (71.9)
Total GPS score, median (IQR)	2 (1–2)
Core comorbidity and scoring of CCI	
Myocardial infarction (score 1)	8 (3.7)
Congestive heart failure (score 1)	5 (2.3)
Cerebral vascular disease (score 1)	7 (3.2)
Peptic ulcer (score 1)	22 (10.1)
Hepatic disease, mild (score 1)	32 (14.7)
Diabetes, mild or moderate, score (1)	20 (9.2)
Pulmonary disease, moderate or severe (score 1)	5 (2.3)
Connective tissue disease (score 1)	9 (4.1)
Diabetes, severe with end-organ damage (score 2)	24 (11.1)
Renal disease, moderate or severe (score 2)	6 (2.8)
Solid tumor, without metastases (score 2)	7 (3.2)
Hepatic disease, moderate or severe (score 3)	6 (2.8)
Solid cancer, with metastases (score 6)	2 (0.9)
Total CCI score, median (IQR)	0 (0–2)
Died before the end of follow up	204 (94.0)

IQR, interquartile range; PPI, Palliative Prognostic Index; ECOG, Eastern Cooperative Oncology Group; GPS, Glasgow Prognostic Score; CRP, C-reactive protein; CCI, Charlson Comorbidity Index.

**Figure 1:**
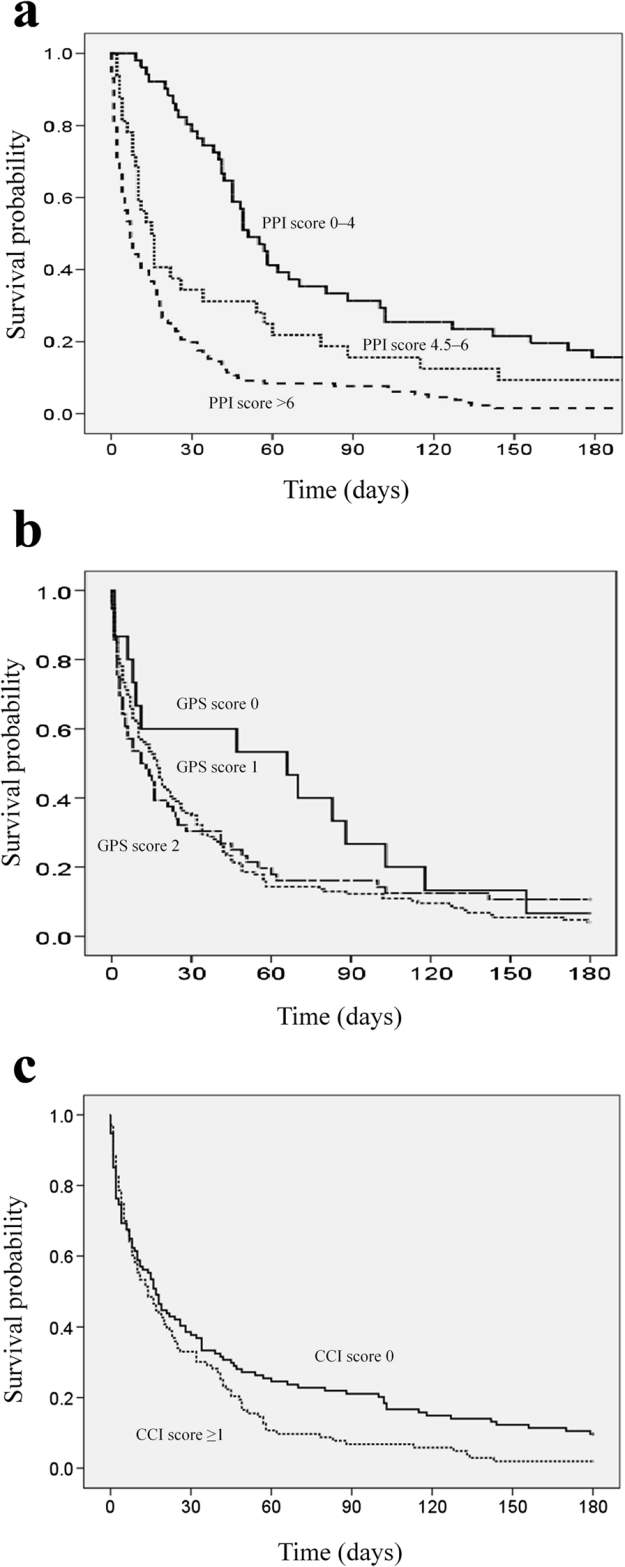
Median patient survival according to (1a) Palliative Prognostic Index (PPI) based good (score 0–4, solid line), intermediate (score 4.5–6, fine dashed line), and poor (score >6, coarse dashed line) prognostic groups; (1b) Glasgow Prognostic Score (GPS) based good (score 0, solid line), intermediate (score 1, coarse dashed line), and poor (score 2, fine dashed line) prognostic groups; (1c) Charlson Comorbidy Index (CCI) based good (score 0, solid line) and poor (score ≥1, fine dashed group) prognostic groups.

The death rate and survival time with respect to PPI, GPS, and CCI categories are given in Table 2. The death rate in the PPI good, intermediate, and poor prognostic groups was 84.6%, 90.9%, and 98.5%, respectively, and the median survival time was 49 days, 15 days, and 7 days (Figure 1a), respectively. The adjusted hazard ration (HR) was 1.73 (95% confidence interval [CI], 1.07–2.80, p = 0.026) when comparing the intermittent and good prognostic groups, and 3.40 (95% CI, 2.31–4.98, p < 0.001) when comparing the poor and good prognostic groups.

**Table 2:** **Survival and death rates based on Palliative Prognostic Index (PPI), Glasgow Prognostic Score (GPS), and Charlson Comorbidity Index (CCI) categories**

**Patient groups and categories**	**No. (%)**	**No. deaths (%)**	**Median survival, days (95% confidence interval)**	**Adjusted hazard ratio (95% confidence interval)**	**P value**
All patients	217(100)	204(94.0)	16 (11.4–21.4)	-	--
PPI					
Good prognosis (score 0–4)	52 (24.0)	44 (84.6)	49 (38.4–59.6)	1	reference
Intermediate prognosis (score 4.5–6.0)	33 (15.2)	30 (90.9)	15 (9.3-20.6)	1.73 (1.07–2.80)	0.026
Poor prognosis (score > 6)	132 (60.8)	130 (98.5)	7 (4.7–9.3)	3.40 (2.31–4.98)	<0.001
GPS					
Good prognosis (score 0)	15 (6.9)	14 (93.3)	66 (0–140.5)	1	reference
Intermediate prognosis (score 1)	56 (30.9)	50 (89.3)	11 (1.5–20.4)	2.12 (1.13–3.97)	0.020
Poor prognosis (score 2)	146 (62.2)	140 (95.9)	17 (12.8–21.2)	1.71 (0.964–3.05)	0.069
CCI					
Good prognosis (score 0)	114 (52.5)	103 (90.4)	17 (12.6–21.4)	1	reference
Poor prognosis (score ≥ 1)	103 (47.5)	101 (98.1)	14 (7.7–20.3)	1.11 (0.82–1.50)	0.51

On the basis of the GPS, 6.9%, 30.9%, and 62.2% of patients were in the good, intermediate, and poor prognostic groups, in which the death rate was 93.3%, 89.3%, and 95.9% and median survival was 66, 11, and 17 days (Figure 1b), respectively. The adjusted HR was 2.12 (95% CI, 1.13–3.97; p = 0.020) when comparing the intermittent and good prognostic groups, and 1.71 (95% CI, 0.96–3.05; p = 0.069) when comparing the poor and good prognostic groups.

The CCI scores assigned 52.5% and 47.5% of patients to the good (CCI = 0) and poor (CCI ≥1) prognostic groups, respectively, in which the death rate was 90.4% and 98.1%, and the median survival time was 17 and 14, respectively (Figure 1c). There was no significant difference in survival between these two patient groups after adjusting for covariates.

**Figure 2:**
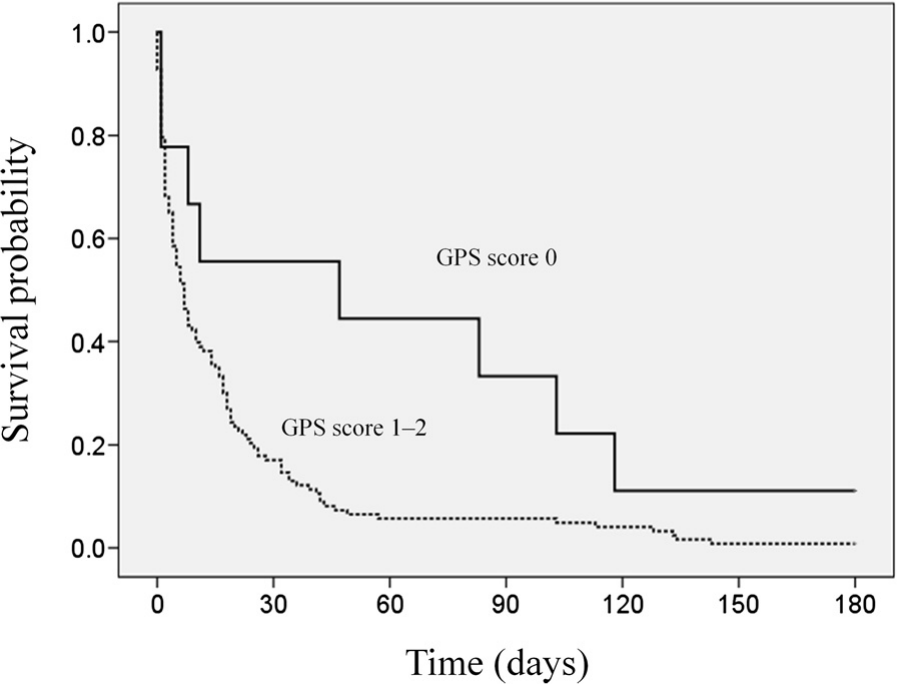
Subgroup survival analysis with respect to Glasgow Prognostic Score (GPS) in the PPI poor prognostic group (solid line, GPS 0; fine dashed line, GPS 1–2).

**Figure 3:**
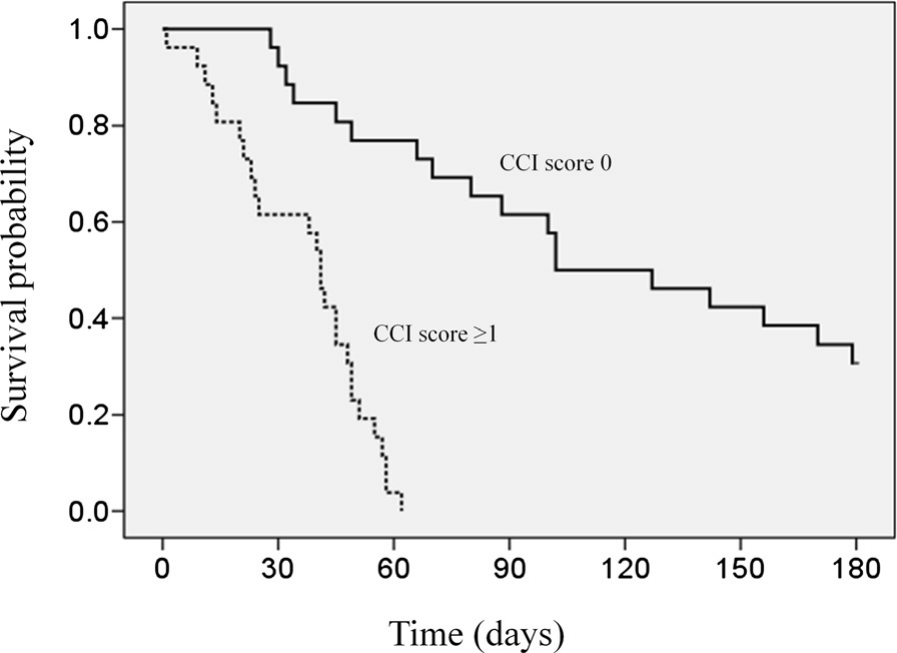
Subgroup survival analysis with respect to Charlson Comorbidity Index (CCI) in the PPI good prognostic group (solid line, CCI score 0; fine dashed line, CCI score ≥1).

Patients categorized into different PPI groups were further stratified by the GPS and CCI scores for survival analysis (Table 3). For patients in the good PPI prognostic group, the death rate was 69.2% and 100%, and the median survival time was 102 and 41 days for those with CCI scores of 0 and ≥ 1, respectively (Figure 2). The adjusted HR was 13.0 (95% CI, 4.7–35.9; p < 0.001) when comparing the CCI score 0 and CCI score ≥ 1 groups. Stratification by the CCI had no effect on survival time for patients in the intermediate or poor PPI prognosis group. For patients in the poor PPI prognostic group, the death rate was 88.9% and 99.2%, and the median survival time was 47 and 7 days for patients with a GPS score 0 and GPS score 1–2, respectively (Figure 3). The adjusted HR was 2.66 (95% CI, 1.23–5.75; p = 0.013) when comparing with a GPS score 0 and score 1–2 groups.

**Table 3:** **Subgroup analysis for survival based on the Glasgow Prognostic Score (GPS), and Charlson Comorbidity Index (CCI) within prognostic groups categorized by the Palliative Prognostic Index (PPI)**

**PPI**	**Subgroup by GPS or CCI**	**No. (%)**	**No. deaths (%)**	**Median survival, days (95% confidence interval)**	**Adjusted HR (95% confidence interval)**	**p**
Good prognosis (total score 0–4)	GPS score 0	4 (1.8)	4 (100)	70 (48.4–91.6)	1	0.62
	GPS score 1-2	48 (22.1)	40 (83.3)	49 (72.8–105.2)	1.32 (0.44–3.97)	
	CCI score 0	26 (12.0)	18 (69.2)	102 (49.5–154.5)	1	<0.001
	CCI score ≥ 1	26 (12.0)	26 (100)	41 (36.0–45.9)	13.0 (4.7–35.9)	
Intermediate prognosis (total score 4.5–6)	GPS score 0	2 (0.9)	2 (100)	6	1	0.22
	GPS score 1–2	31 (14.3)	28 (90.3)	16 (10.6–21.4)	0.36 (0.07–1.88)	
	CCI score 0	19 (8.8)	17 (89.5)	16 (0–31.6)	1	0.80
	CCI score ≥1	14 (6.5)	13 (92.9)	10 (7.5–12.4)	1.14 (0.40–3.26)	
Poor prognosis (total score >6)	GPS score 0	9 (4.1)	8 (88.9)	47 (0–152)	1	0.013
	GPS score 1–2	123 (56.7)	122 (99.2)	7 (4.7–9.3)	2.66 (1.23–5.75)	
	CCI score 0	69 (31.8)	68 (98.6)	7 (1.9–12.1)	1	0.21
	CCI score ≥1	63 (29.0)	62 (98.4)	7 (4.1–9.9)	0.72 (0.49–1.07)

## Discussion

Our findings show that the PPI, but not the GPS or CCI, have significant predictive value for the life expectancy of terminally ill patients with hematologic malignancies under palliative care. To the best of our knowledge, this study was the first to establish the PPI as a prognosticator of life expectancy in these patients. Adding the CCI in good the PPI prognostic group helps to identify patients likely to have less favorable outcomes; while adding the GPS in the poor PPI prognostic group increases the accuracy to distinguish those less likely to have the poorest outcomes. The use of these tools might enable health workers to provide more appropriate end-of-life care for the patient, and to refer patients to palliative care earlier. Patients and their families might then also have adequate time to discuss end-of-life issues, and to prepare for the patient’s death.

The PPI assessment for predicting survival had two major limitations. First, the PPI was calculated for patients immediately after palliative care was imitated, thus failing to account for subsequent changes in the patient’s condition or their clinical course. Therefore, a single PPI score may not be an appropriate predictor of long-term survival in terminally ill patients. The combination of the initial PPI with subsequent changes in the PPI might solve this problem [17,18]. The second difficulty is that the PPI is based only on clinical features (performance status, dyspnea, oral intake, edema and delirium) without regard to comorbidity and nutritional status. Dying patients, regardless of their underlying diseases, often had clusters of PPI symptoms [19]. Identifying these core symptoms can help clinicians estimate a patient’s life expectancy. The PPI is simple to use for this reason, but also has limited clinical application because it is difficult to make further survival predictions for patients with similar scores. Moreover, it is almost impossible to further stratify patients in the good PPI prognostic group because they may be virtually free of any of these core symptoms. By combining it with the CCI and GPS, as in the present study, a better discrimination of survival in patients categorized into the same prognostic group by the PPI is possible.

The CRP is an acute-phase protein recognized as a marker of systemic inflammation [20]. Albumin represents a marker of nutritional status and hypoalbuminemia might reflect malnutrition or declining health status [21]. The GPS, based on CRP and albumin levels, has been demonstrated to have prognostic value in a wide range of solid cancers since it was first proposed in 2003 [22]. There were significant differences in survival among GPS scores of 0, 1 and 2 in patients with metastatic colorectal cancers treated with bevacizumab or anti-EGFR therapy [23,24]. The prognostic value of the GPS in other hematologic malignancies or in patients with a hematologic malignancy under palliative care has never been investigated, and ours is the first study to assess the latter. Although our findings show that the GPS was an independent prognostic factor when comparing the score 0 and score 1 groups, the difference in survival between the score 0 and score 2 groups was only of borderline significance (p = 0.069). This may be because the statistical analysis was limited by the small number of patients, as only 6.9% of patients were in the GPS score 0 group. In addition, there was no significant difference in survival between patients of GPS 1 and 2. This may be due to the very short overall survival (median survival of 16 days) in these groups. Importantly, our study showed that low CRP levels and normal albumin levels are significant positive prognostic factors, especially in patients presenting with multiple negative clinical features (PPI score > 6).

The CCI is the most commonly used comorbidity index [7-12]. Initially, the CCI was to assess the role of comorbidity on mortality risks in longitudinal studies and has since been shown to be associated with a poor outcome in patients with hematologic malignancies who are undergoing hematopoietic stem cell transplantation [9] or antitumor therapy [10-12]. CCI is also associated with more frequent hospital mortality in patients with hematologic malignancies who were admitted to the intensive care unit [25]. We previously showed that patients with hematologic malignancies were younger than those with solid cancers [1]. This may explains why more than half of our patients presented with a CCI score 0. In order to evaluate how comorbidities impacted survival, we used a modified CCI [9], excluding age and hematologic malignancies from its original designation [26]. We showed that comorbidities had little impact on survival in terminally ill hematologic malignancies patients. However, CCI may still be a prognosticator in patients in the good PPI prognostic group.

The strength of this study was in the large number of patients with a hematologic malignancy under palliative care. However, this was a retrospective, single-institute study, and the timing of the CRP and albumin measurements was not planned in advance, thereby introducing the possibility of bias. The prognostic value of the PPI, GPS, and CCI in terminally ill patients with hematologic malignancies under or not under palliative care needs further prospective exploration and validation.

## Conclusions

PPI is a useful prognosticator of life expectancy in terminally ill patients with a hematologic malignancy under palliative care. Concurrent use of the GPS and CCI improved the accuracy of prognostication using the PPI.

## Abbreviations

PPI: Palliative prognostic index
CCI: Charlson comorbidity index (CCI)
GPS: Glasgow prognostic score
CRP: C-reactive protein
PCCS: Palliative care consultation service
ECOG PS: Eastern cooperative oncology group performance status
PPS: Palliative performance scale
IQR: Interquartile range
HR: Hazard ratios

## References

[1] Hung YS, Wu JH, Chang H, Wang PN, Kao CY, Wang HM (2013). Characteristics of patients with hematologic malignancies who received palliative care consultation services in a medical center. Am J Hosp Palliat Care.

[2] Health Registry Annual Report 2009, Republic of China (2011). Republic of China: Bureau of Health Promotion, Department of Health, executive Yuan, Republic of China.

[3] Morita T, Tsunoda J, Inoue S, Chihara S (1999). The Palliative Prognostic Index: a scoring system for survival prediction of terminally ill cancer patients. Support Care Cancer.

[4] Stone CA, Tiernan E, Dooley BA (2008). Prospective validation of the palliative prognostic index in patients with cancer. J Pain Symptom Manage.

[5] Maltoni M, Scarpi E, Pittureri C, Martini F, Montanari L, Amaducci E (2012). Prospective comparison of prognostic scores in palliative care cancer populations. Oncologist.

[6] Cheng WH, Kao CY, Hung YS, Su PJ, Hsieh CH, Chen JS (2012). Validation of a palliative prognostic index to predict life expectancy for terminally ill cancer patients in a hospice consultation setting in Taiwan. Asian Pacific J Cancer Prev.

[7] Newschaffer CJ, Bush TL, Penberthy LE, Bellantoni M, Helzlsour K, Diener-West M (1998). Does comorbid disease interact with cancer? An epidemiologic analysis of mortality in a cohort of elderly breast cancer patients. J Gerontol A Biol Sci Med Sci.

[8] Extermann M, Overcash J, Lyman GH, Parr J, Balducci L (1998). Comorbidity and functional status are independent in older cancer patients. J Clin Oncol.

[9] Sorror ML, Maris MB, Storer B, Sandmaier BM, Diaconescu R, Flowers C (2004). Comparing morbidity and mortality of HLA-matched unrelated donor hematopoietic cell transplantation after nonmyeloablative and myeloablative conditioning: influence of pretransplantation comorbidities. Blood.

[10] Wieringa A, Boslooper K, Hoogendoorn M, Joosten P, Beerden T, Storm H (2014). Comorbidity is an independent prognostic factor in patients with advanced-stage diffuse large B-cell lymphoma treated with R-CHOP: a population-based cohort study. Br J Haematol.

[11] Breccia M, Frustaci AM, Cannella L, Stefanizzi C, Latagliata R, Cartoni C (2009). Comorbidities and FLT3-ITD abnormalities as independent prognostic indicators of survival in elderly acute myeloid leukaemia patients. Hematol Oncol.

[12] Lin TL, Kuo MC, Shih LY, Dunn P, Wang PN, Wu JH (2012). The impact of age, Charlson comorbidity index, and performance status on treatment of elderly patients with diffuse large B cell lymphoma. Ann Hematol.

[13] McMillan DC (2013). The systemic inflammation-based Glasgow Prognostic Score: a decade of experience in patients with cancer. Cancer Treat Rev.

[14] Bureau of National Health Insurance, Taiwan, Republic of China. Symptom record form in patients with terminal illness disease for hospice consultation care nurse specialist. Available from http://www.nhi.gov.tw/information/bbs_detail.aspx?bulletin_ID¼1404&menu¼9&menu_id¼545. Accessed May 08, 2015.

[15] Anderson F, Downing GM, Hill J, Casorso L, Lerch N (1996). Palliative Performance Scale (PPS): a new tool. J Palliat Care.

[16] Ma C, Bandukwala S, Burman D, Bryson J, Seccareccia D, Banerjee S (2010). Interconversion of three measures of performance status: an empirical analysis. Eur J Cancer.

[17] Kao CY, Hung YS, Wang HM, Chen JS, Chin TL, Lu CY (2014). Combination of initial palliative prognostic index and score change provides a better prognostic value for terminally ill cancer patients: a six year observational cohort study. J Pain Symptom Manage.

[18] Hung CY, Wang HM, Kao CY, Lin YC, Chen JS, Hung YS (2014). Magnitude of score change for the palliative prognostic index for survival prediction in patients with poor prognostic terminal cancer. Support Care Cancer.

[19] Viganò A, Bruera E, Suarez-Almazor ME (1999). Terminal cancer syndrome: myth or reality ?. J Palliat Care.

[20] Marnell L, Mold C, Du Clos TW (2005). C-reactive protein: Ligands, receptors and role in inflammation. Clin Immunol.

[21] McMillan DC (2009). Systemic inflammation, nutritional status and survival in patients with cancer. Curr Opin Clin Nutr Metab Care.

[22] Forrest LM, McMillan DC, McArdle CS, Angerson WJ, Dunlop DJ (2003). Evaluation of cumulative prognostic scores based on the systemic inflammatory response in patients with inoperable non-small-cell lung cancer. Br J Cancer.

[23] Maillet M, Dréanic J, Dhooge M, Mir O, Brezault C, Goldwasser F (2014). The predictive and prognostic value of the Glasgow Prognostic Score in metastatic colorectal carcinoma patients receiving bevacizumab. Anticancer Drugs.

[24] Dréanic J, Maillet M, Dhooge M, Mir O, Brezault C, Goldwasser F (2013). Prognostic value of the Glasgow Prognostic Score in metastatic colorectal cancer in the era of anti-EGFR therapies. Med Oncol.

[25] Azoulay E, Mokart D, Pène F, Lambert J, Kouatchet A, Mayaux J (2013). Outcomes of critically ill patients with hematologic malignancies: prospective multicenter data from France and Belgium–a groupe de recherche respiratoire en réanimation onco-hématologique study. J Clin Oncol.

[26] Charlson ME, Pompei P, Ales KL, MacKenzie CR (1987). A new method of classifying prognostic comorbidity in longitudinal studies: Development and validation. J Chronic Dis.

